# Effects of simultaneously performed cognitive and physical training in older adults

**DOI:** 10.1186/1471-2202-14-103

**Published:** 2013-09-23

**Authors:** Nathan Theill, Vera Schumacher, Rolf Adelsberger, Mike Martin, Lutz Jäncke

**Affiliations:** 1 Division of Gerontopsychology, Department of Psychology, University of Zurich, Zurich, Switzerland; 2 International Normal Aging and Plasticity Imaging Center (INAPIC), University of Zurich, Zurich, Switzerland; 3 Department of Social Work, Zurich University of Applied Sciences, Zurich, Switzerland; 4 Department of Information Technology and Electrical Engineering, Federal Institute of Technology (ETH), Zurich, Switzerland; 5 Division of Neuropsychology, Department of Psychology, University of Zurich, Zurich, Switzerland; 6 University Research Priority Program “Dynamics of Healthy Aging”, University of Zurich, Zurich, Switzerland

**Keywords:** Cognitive training, Physical training, Simultaneous training, Motor-cognitive dual task training, Working memory, Brain plasticity

## Abstract

**Background:**

While many studies confirm the positive effect of cognitive and physical training on cognitive performance of older adults, only little is known about the effects of simultaneously performed cognitive and physical training. In the current study, older adults simultaneously performed a verbal working memory and a cardiovascular training to improve cognitive and motor-cognitive dual task performance. Twenty training sessions of 30 minutes each were conducted over a period of ten weeks, with a test session before, in the middle, and after the training. Training gains were tested in measures of selective attention, paired-associates learning, executive control, reasoning, memory span, information processing speed, and motor-cognitive dual task performance in the form of walking and simultaneously performing a working memory task.

**Results:**

Sixty-three participants with a mean age of 71.8 ± 4.9 years (range 65 to 84) either performed the simultaneous training (*N =* 21), performed a single working memory training (*N* = 16), or attended no training at all (*N* = 26). The results indicate similar training progress and larger improvements in the executive control task for both training groups when compared to the passive control group. In addition, the simultaneous training resulted in larger improvements compared to the single cognitive training in the paired-associates task and was able to reduce the step-to-step variability during the motor-cognitive dual task when compared to the single cognitive training and the passive control group.

**Conclusions:**

The simultaneous training of cognitive and physical abilities presents a promising training concept to improve cognitive and motor-cognitive dual task performance, offering greater potential on daily life functioning, which usually involves the recruitment of multiple abilities and resources rather than a single one.

## Background

Both cognitive and physical trainings have been successfully applied to improve cognitive performance in old age. Although cognitive trainings typically improve the targeted ability [1,2], some of them lead to a transfer of improvements to tasks that have not been explicitly trained [3,4]. In particular, training of working memory has been shown to be effective in old age with respect to a variety of abilities such as visuospatial working memory, block span or reading span tasks, inhibition, processing speed, fluid intelligence, visual episodic memory or verbal learning [5-8]. In comparison, physical training, especially in the form of cardiovascular stimulating activity, increases cognitive performance in almost all abilities [9-11].

Since both cognitive and physical trainings result in improvements in cognitive abilities, there might be some shared underlying mechanisms that lead to these changes in cognitive performance. As neuroplasticity of the brain is supposed to provide the basis for substantial behavioral changes, the question arises whether specific mechanisms exist that are essential for transfer of training effects. On one hand, training could be most effective if it recruits the maximum of different brain regions to induce functional or structural changes. On the other hand, brain regions known to be involved in higher-order cognition could promote interconnections with other regions that lead to the observable behavioral changes. It has to be determined how exactly cognitive activities such as engaging in working memory tasks and physical activities affect brain functioning, and which different, shared, or complementary mechanisms exist that result in improvements in cognitive performance. In fact, working memory training as well as physical training have been shown to induce functional changes in brain regions of older adults involved in higher-order cognition, such as the prefrontal cortex (PFC) or the parietal cortex [11-15]. The PFC is crucial for performing complex tasks involving planning and cognitive control [16,17] and, thus, is also activated when performing working memory tasks [18,19]. However, whereas cognitive activity usually involves specific brain regions such as PCF, global as well as localized effects are assumed for physical exercise [20]. Physical exercise is associated with higher cerebral blood flow [21-23], as well as with the release of brain-derived neurotrophic factor (BDNF) and insulin growth factor (IGF-1), both of which are assumed to be involved in synaptogenesis, angiogenesis, and neurogenesis, i.e., the main underlying mechanisms for neural plasticity [24-26]. In addition, physical exercise is reported to result in structural changes of increased gray or white matter volume in many different regions such as the PFC, the hippocampus, the motor cortex, the temporal cortex or the cerebellum [20,27-29].

According to these findings, there are overlapping as well as distinct effects of cognitive and physical training. As a result, cognitive and physical activities could have some complementary effects that are able to optimize training output on cognition. Consequently, combining cognitive and physical training should provide additional effects that go beyond the effects of training the single underlying components. However, so far only a few studies have combined cognitive with physical training. Although they did not specifically focus on the training of working memory, these studies can still provide information about the effects of combining cognitive and physical training interventions in older adults. Two studies showed larger improvements for a combined cognitive and physical training compared to cognitive or physical training alone [30,31]. A third study found only a general advantage of both a combined cognitive and physical training, as well as physical exercise training when compared to a control group, but no superior effect of the combined training condition over the exercise condition [32].

One important limitation of previous training studies is that the trainings were conducted sequentially. As a consequence, potential synergistic effects are neglected, which could emerge when performing both trainings simultaneously. At least, it is imaginable that immediate effects during physical activity such as increased cerebral blood flow boost the effect of an additional stimulation through a cognitive task and promote interconnections within and between different brain regions. Although there are so far no data confirming this theory, acute physical exercise has been shown to be associated with improved performance as well as enhanced PFC activity during a subsequent working memory or executive function task in both younger and older adults [33,34]. These findings indicate increased task-related blood flow in corresponding brain areas during physical arousal, which could not only promote the performance during the present task, but also training gain and transfer.

In addition, physical activities are mostly performed in the context of specific perceptual and cognitive demands. Thus, the simultaneous performance of physical and cognitive tasks requires the integration of two different tasks, representing a cognitively demanding dual task, activating not only those networks involved in controlling each task, but also networks being only active or showing increased activity under demanding dual task conditions, e.g., PFC, inferior parietal cortex, dorsal PFC, and right inferior frontal gyrus [35-38]. In line with this, an increased neuronal efficiency as well as increased dual task performance has been found following cognitive dual task training [39]. In the context of motor-cognitive dual task training, a recent study found strengthened connectivity in brain regions within the cerebellum that are supposed to integrate motor and cognitive network [40]. As a result, dual task performance can be specifically trained, and has been shown to even transfer to other dual task situations [41,42]. Consequently, a substantial additional benefit is expected when performing a cognitive and a physical task simultaneously.

In the current study, older adults were trained with a simultaneous verbal working memory and cardiovascular treadmill training. The training was compared to single working memory training and a passive control group. In line with previous research and in reference to the framework for plasticity from Lövdén, Bäckman, Lindenberger, Schaefer and Schmiedek [43], both the simultaneous and the single cognitive training were adaptive with a continuously adjusted task difficulty as a function of the individual performance level. Adaptive working memory training has been shown to be more effective with regard to both behavioral as well as neuronal changes compared to non-adaptive working memory training [6,12]. Training gains were expected in cognitive as well as motor-cognitive dual task performance. For that reason, the participants were tested with respect to their cognitive performance in selective attention, paired-associates learning, executive control, reasoning, memory span, and information processing speed. Motor-cognitive dual task performance was assessed with a simultaneous walking and working memory task. Older individuals usually adapt to a demanding motor-cognitive dual task situation by reducing their performance of at least one of the underlying tasks. During this specific task, this adaptation is reflected by lower performance in the working memory task and lower walking speed and regularly compared to the performance of one of the tasks alone [44,45]. Therefore, increased motor-cognitive dual task performance should be expressed by improved performance in the working memory task and gait parameters under dual task as well as by lower reduction of gait parameters from single to dual task walking.

As a conclusion, the following hypotheses were formulated: 1. Both training groups were expected to improve their performance in the two training tasks over the course of the training. 2. The participants of both training groups were expected to demonstrate training gains in the cognitive transfer tasks when compared to the passive control group, with larger improvements for the simultaneous training group. 3. The simultaneous training group was expected to improve in motor-cognitive performance compared to the single cognitive training group and passive control group, with no substantial differences between the single cognitive training and the control group.

## Methods

### Participants

Sixty-three healthy older adults with a mean age of 71.8 ± 4.9 (range 65 to 84) participated in the study, 46 (73%) of them were females. Participants were recruited through (a) advertisement in local newspapers, (b) a call at the senior University of Zurich, and (c) draws from the participant pool of the Division of Gerontopsychology of the Department of Psychology of the University of Zurich. The study was approved by the ethics committee of the Faculty of Arts of the University of Zurich. Participants signed up either for the simultaneous training condition (*N* = 21), the single cognitive training condition (*N* = 16), or the passive control group (*N* = 26). They did not get any payment or refund of their travelling expenses but a detailed feedback of their performance and training progress at the end of the study. At the beginning of the study and after informed consent was obtained, the participants were screened for cognitive impairment and medical conditions. One participant of the control group had to be excluded due to reading disability. Another eleven individuals did not complete the study mostly due to time constraints or illness during the course of the study, so there were 51 individuals left who fulfilled all inclusion criteria and completed the study. Characteristics of those participants are displayed on Table 1. However, each group was affected similarly with three individuals in the simultaneous training group and four individuals in the single cognitive training and the passive control group, respectively, who dropped out during the course of the study. Furthermore, there were no significant differences in demographic data, cognitive status, baseline scores of cognitive tasks, and training progress between those who completed the study and those who dropped out (all *p* > 0.05).

**Table 1:** Demographic characteristics of the participants included in the study

	**Simultaneous training (*N*** **= 18)**	**Single cognitive training (*N*** **= 12)**	**Control group (*N*** **= 21)**	
**Variable**	***M *( *SD *)**	***M *( *SD *)**	***M *( *SD *)**	***p***
Age	72.39 (4.19)	73.33 (6.08)	70.90 (4.77)	.369
MMSE	28.94 (1.00)	29.25 (0.87)	29.24 (0.89)	.550
Education (years)	13.76 (2.95)	14.92 (4.93)	13.18 (2.87)	.394
Activity (MET)	112.10 (54.85)	159.57 (36.07)	147.02 (73.57)	.036

*Notes*. MMSE = Mini Mental State Examination, MET = Metabolic Equivalent.

### Materials

#### ***Cognitive transfer tasks***

The test battery consisted of six computer-based tests to assess the performance in the following domains: selective attention, paired-associates learning, executive control, reasoning, memory span, and information processing speed. Three parallel versions of the tests were programmed using E-Prime 2.0 Professional (Psychology Software Tools, Pittsburgh, PA), one for each test session. Due to the limited test material of the reasoning task, the posttest contained the same task as the pretest. The tests were always preceded by an instruction and an example or trial. All tests were presented on a 24-inch monitor using a standard wireless keyboard as input device.

*Selective Attention* was measured using the continuous performance task adapted from [46]. At this task, letters were presented consecutively in the middle of the screen. The objective was to correctly identify if the letter X followed the letter A, which had to be signaled by pressing the button ′2‵ on the keyboard. For all other characters or the letter X following another letter than the letter A, the participants had to press the button ′1‵ on the keyboard. Each letter was presented for 300 milliseconds with an interval of 4.9 seconds between the probe and the target letter and 2 seconds between the target and the following probe letter. There was an exercise run followed by two consecutive blocks of 30 pairs of letters each. The total number of correct answers as well as the number of correct answers depending on the combinations A-X (X following A), B-X (X following any other letter than A) or A-Y (any other letter than X followed A) were calculated for the analysis.

*Paired-associates learning*: At this test, the participants learned a sequence of seven combinations of shapes and colors (adapted from [47]). Each combination was presented in a random order for 4 seconds. Subsequently, the target shape was represented at the top of the screen with a choice of eight colors listed below. Participants had to press the button of the color that had been presented with the target shape during learning phase.

*Executive control*: During this test, up to nine pictures were presented with different numbers of circles and triangles on each picture (adapted from [47]). Participants were instructed to alternately count either the circles or the triangles. They had 60 seconds to consider as many pictures as possible and to learn the sequence of the numbers of the counted shapes, which made a total sequence of at most nine digits. Subsequently, the participants had 30 seconds to type in this sequence of numbers on the keyboard. The test consisted of three single trials each containing the same nine pictures in different order, so the participants could achieve a maximum score of 27.

*Reasoning* was measured with the Standard Progressive Matrices test [48]. Matrices of different shapes or patterns were presented, in which a part was missing. The objective was to find out which piece of a given list could complete these matrices meaningfully. The participants had five minutes time to do as many matrices as possible out of a set of 27 pictures.

*Memory span* was examined with the operation span test adapted from [49]. During this test, the participants had to learn different sequences of words out of a pool of 100 words, varying between two and six words per trial. Participants were instructed to learn the words in the given order. Between these words, correct or incorrect mathematical operations were displayed and participants had to respond by pressing either the button “j” for correct or “n” for incorrect on the keyboard. After each trial, participants were requested to type in the word in the correct order. To make sure that the distraction task was done properly, only individuals who scored more than 85% in the calculation task were included in the analysis. Thirty-nine of the 51 participants who completed the study fulfilled this criterion. Of these, the number of correctly recalled words at correct serial position was calculated for all trials see [49].

*Information processing speed* was measured using a digit-letter task similar to the Digit Symbol Substitution Task from Wechsler [50], except that participants were required to assign digits to letters instead of symbols to digits. At this task, five combinations of digits and letters were presented on the upper part of the screen, with one of the letters additionally being displayed as a probe on the lower part of the screen. The objective was to type the corresponding number as fast as possible. The task lasted 90 seconds and the total number of correct answers during this time was calculated.

#### ***Motor-cognitive dual task***

During the motor-cognitive dual task, gait performance was investigated under single- and dual-task conditions while performing a working memory task. The participants walked at their normal, self-selected speed over a distance of 20 meters, with a turning point at a cone after 10 meters. An additional distance of 1.5 meters was arranged at the beginning and the end of the track for the accelerating and decelerating phase. Under dual task condition, the participants additionally performed a working memory task by counting backwards in steps of seven, beginning alternately with either 501, 502, or 503. The correct steps of calculations and errors were noted. Time for walking was measured to determine gait velocity and gait patterns were assessed using four acceleration sensors located at upper and lower legs that communicate wireless to a computer via a portable device worn as a belt (for details see [51]). Gait data were recorded with a scanning frequency of 25 Hz and gait parameters were reproduced in step duration. Gait variability then was calculated dividing the standard deviation of step duration with the mean time for step duration multiplied by hundred (step-to-step variability).

#### ***Verbal working memory training***

The verbal working memory training session contained a computer-based n-back training (adapted from [52]) and serial position training, each of them lasting approximately 15 minutes. The training was programmed with E-Prime 2.0 Professional (Psychology Software Tools, Pittsburgh, PA) and was displayed at a 24-inch monitor using a standard wireless mouse as input device. During the n-back training, the participants had to continuously respond to a series of letters appearing all three seconds on the computer screen, always comparing the subsequent letters with the letter in a given sequence before (n-back). The letters were presented for 500 milliseconds each and participants had to press the mouse button either once for any new letter or twice for any letter matching the one n positions before. One trial consisted of 30 letters and lasted 90 seconds. During the serial position training, participants had to learn a sequence of words in the correct order, each of them presented for three seconds. The learning phase was followed by a distraction phase in which participants had to decide if words out of a series of three words were meaningful or not. At the end, the words of the learning phase were presented in either the correct or incorrect word order. Again, they had to respond by pressing the mouse button either once if the sequence of the words had changed or twice if the sequence was the same.

Both tasks were adaptive, which means that the difficulty level rose with increasing performance. The n-back training started with 1-back and the serial position training started with a sequence of three words. The difficulty level gradually increased whenever participants achieved 80 percent within a level. As soon as the participants achieved level six for the first time, a new training session started with three levels below the level they achieved at the end of the last training session. During the n-back task, the participants also could fall back if their performance was less than 60 percent within a level. During the serial position task, they stayed at their latest level as long as they did not achieve at least 80 percent and a new training session started one level below the level they achieved during the last training session. Progress of training was determined by maximum level achieved during the training session.

#### ***Physical training***

The participants walked quickly on a treadmill for about 40 minutes including a warm-up period at a self-selected speed. The treadmill training was pulse monitored to make sure that the pulse rate was in an aerobic range of at least 60 percent and at most 80 percent of their age-related maximum value (heart rate of 220 minus age) and the speed of the treadmill was adapted in case the heart rate went below or beyond this range. For safety reasons, the participants were fastened with a special safety belt that was ceiling-mounted above the treadmill to prevent them from falls in case they lost balance or stumbled.

#### ***Procedure***

The participants of the training groups attended to 20 training sessions and three test sessions overall, whereas the participants of the control group only took part in these test sessions. The tests were conducted before the training (pretest), in the middle of the training after five weeks (interim test), and after the completion of the training (posttest). The participants of the control group performed the tests at the same time intervals. The test sessions lasted between one and a half and two hours and started with the cognitive test battery, followed by the motor-cognitive dual task. The participants received an introduction to the test process and conditions, and to the handling of the particular tests. They were allowed to ask questions during the cognitive tests in case they did not understand the instructions. During the motor-cognitive dual task, the participants were first asked to walk at their normal, self-selected speed. Subsequently, they were asked to walk the same distance and count backward in steps of seven from either 501, 502 or 503. They were not instructed to prioritize any of the tasks.

At the first test session, the test procedure also involved a demographic and medical conditions questionnaire, and the screening for cognitive impairment Mini Mental State Examination (MMSE), [53]. In addition, the participants of the simultaneous training condition walked on the treadmill for 10 minutes at the end of the first test session to habituate to the treadmill and to determine their optimal walking speed for the subsequent training sessions. After the test session, the German-PAQ-50+ [54] was filled out at home. This questionnaire is designed to estimate the physical activity of older adults based on different activities, including sport, housework, yard work, job, and leisure activities. Depending on the effort of a specific activity, different scores of metabolic equivalent (MET-scores) are multiplied with the number of hours spent for this activity. These scores are then added together to give a total score of metabolic equivalent (MET).

The trainings were conducted in two sequences of 10 training sessions held twice weekly over a period of 10 weeks. Both the single condition and simultaneous training groups received a detailed instruction to the cognitive training program before the first training session, including a trial run of both cognitive trainings.

The participants of the simultaneous group then performed the cognitive training on a computer screen located at eye level approximately one meter in front of them while simultaneously walking on the treadmill. The single-condition training group performed the same cognitive training tasks while sitting at the table. Both training groups performed the training on the same type of computer and monitor using a wireless mouse as input device.

#### ***Statistical analysis***

Results of the metabolic equivalent (MET) score of the physical activity questionnaire and dual task gait variability were transformed for calculations with natural logarithmic transformation to eliminate outliers. Baseline performance and differences in demographic data between the groups were compared with Analysis of Variance (ANOVA) and motor-cognitive dual task performance changes from single to dual task were calculated using Analysis of Variance for Repeated Measures (ANOVAR).

The training progress was analyzed with linear mixed-effects models (LMM). The random intercept model was calculated first as baseline model. Subsequently, the fixed and random factors for slope (training progress) were added, and eventually the fixed factor for group and the interaction between group and training. Goodness of fit indices between the models were compared using -2log likelihood (-2LL), and Chi-square tests were calculated to select the model with the optimal fit. ANOVA and LMM were also used to compare demographic data, baseline performance, and training progress of the participants who completed the study with those who dropped out during the course of the study.

Training benefits to the cognitive transfer tasks as well as motor-cognitive dual task were analyzed using multiple regression analysis with planned comparisons involving orthogonal contrast and polynomial trend coding. Contrast coding variables were created according to the hypotheses. For the cognitive transfer tasks, the control group was first contrasted against the average of both training groups with a subsequent comparison between the two training conditions to analyze the advantage of the simultaneous training compared to the single cognitive training. For the motor-cognitive dual task, the simultaneous training group was contrasted against the average of the single cognitive training and the control group with a subsequent comparison between the single cognitive training and the control condition. Based on the study design with three points of measurement, polynomial trend coding variables were created for the linear and quadratic trend. To account for subject effects, effect code variables were defined for the individuals within each group. For the transfer tasks, a significant interaction between both the first and the second contrasts and the linear trend was expected in order to illustrate the expected linear training effects for the two training conditions. For the motor-cognitive dual task, a significant interaction was expected between the first contrast and the linear trend but no significant interaction between the second contrast and the linear trend to illustrate the advantage of the simultaneous training compared to both the single cognitive training and the control condition. Statistics were calculated using SPSS 20 for Macintosh (SPSS Inc., Chicago, Il.) with a significance level of α = .05.

## Results

Due to technical problems, the results of the selective attention task of only 39 participants and gait variability of only 42 participants were analyzed. Data of the executive control and memory span task from two individuals had to be excluded due to unauthorized note taking during the tests. In addition, gait parameters of one individual were excluded because of the large deviations in both gait velocity and variability.

### Baseline characteristics and performance

The groups did not differ with respect to their demographic data such as age or education, as well as to their scores in the MMSE (all *p >* .05, Table 1). In addition, they did not show any differences in their baseline performance either in the cognitive test battery or in gait velocity, gait variability or working memory performance in the motor-cognitive dual task (all *p* > .05). However, the groups differed in the metabolic equivalent (MET) score of the physical activity questionnaire (*p* = .036).

### Training progress

**Figure 1:**
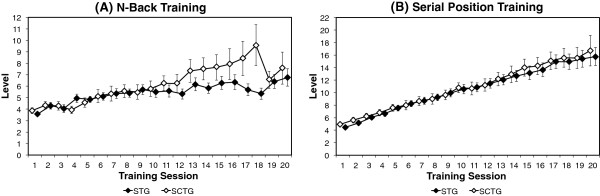
**Training progress during the n-back (A) and serial position (B) training.** The participants of the simultaneous training group (STG) and single cognitive training group (SCTG) significantly improved their performance during both trainings (*p <* .001), but with no differences in training progress between the two training groups. Bars represent ± standard error of the mean.

For both trainings, the linear mixed model with the best fit was the model with fixed effects for intercept and training as well as random effects for intercept and slope (training) with a variance components covariance structure, but without fixed effects for group or interaction between group and training (Table 2 and 3). The participants of both training groups thus showed a significant increase in the n-back (*F* (1,34.46) = 22.34, *p <* .001) as well as the serial position training tasks (*F* (1,34.47) = 208.59, *p <* .001), with no significant group differences in terms of intercept and change over time, which means that the two training groups did not differ in their training progress either in the n-back or the serial position training. Training curves for both training groups are displayed in Figure 1.

**Table 2:** Model fit of the linear mixed-effects models for the training progress during the n-back training

**Model**	***-2LL***	***Δdf***	***Δ-2LL***	**AIC**	**BIC**
Model 0	2622.67		-	2628.67	2642.01
Model 1	2427.32	1	195.35***	2435.32	2453.11
Model 2	2096.25	1	331.07***	2106.25	2128.48
Model 3	2093.12	2	1.80	2107.12	2138.25

*Notes*. -2LL = -2Log Likelihood, Δdf = Change of degrees of freedom between the models, Δ-2LL = Change of -2Log Likelihood between the models, AIC = Akaike’s information criterion, BIC = Bayesian information criterion of Schwarz. Model 0 = random intercept model, Model 1 = additional fixed effect for slope, Model 2 = additional random effect for slope, Model 3 = additional fixed effects for group and interaction between group and slope.

*** < .001.

**Table 3:** Model fit of the linear mixed-effects models for the training progress during the serial position training

**Model**	***-2LL***	***Δdf***	***Δ-2LL***	**AIC**	**BIC**
Model 0	3538.07		-	3544.07	3557.41
Model 1	2491.72	1	1046.35***	2499.72	2517.51
Model 2	1636.05	1	855.67***	1646.05	1668.28
Model 3	1635.47	2	0.58	1649.47	1680.60

*Notes*. -2LL = -2Log Likelihood, Δdf = Change of degrees of freedom between the models, Δ-2LL = Change of -2Log Likelihood between the models, AIC = Akaike’s information criterion, BIC = Bayesian information criterion of Schwarz. Model 0 = random intercept model, Model 1 = additional fixed effect for slope, Model 2 = additional random effect for slope, Model 3 = additional fixed effects for group and interaction between group and slope.

*** < .001.

### Cognitive transfer tasks

**Figure 2:**
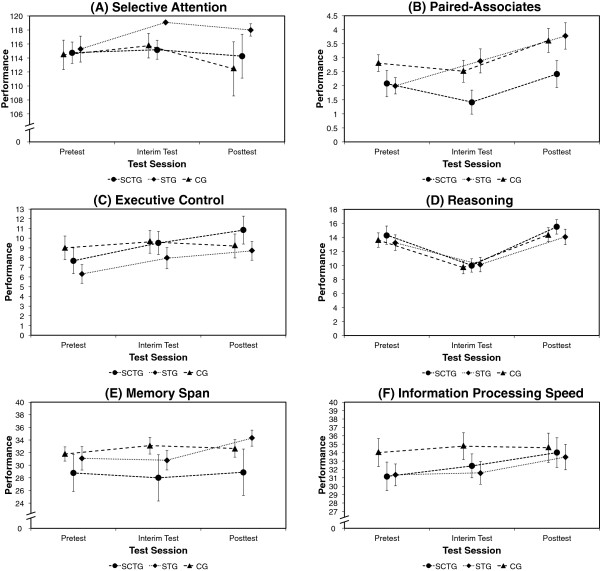
**Performance in the cognitive transfer tasks (A-F).** The participants of the simultaneous training group (STG) and single cognitive training group (SCTG) showed a larger improvement in the executive control task when compared to the control group (CG) (*p* = .037), with no differences between the two training conditions. In addition, the combined training group showed larger training gains in the paired-associates task compared to the single cognitive training group (*p* = .018). Bars represent ± standard error of the mean.

Training gains on cognitive transfer tasks are displayed in Figure 2. Compared to the control group, there was a significant linear improvement in the executive control task (*F* (1,92) = 3.284, *p* = .037, *R*^2^ = .011) as a result of the training, but with no differences between the two training conditions (*F* (1,92) = 0.178, *p* = .337, *R*^2^ = .001), indicated by a significant interaction between the first group contrast and the linear trend (Table 4). There was a positive but not significant trend for the training gain of both training groups (*F* (1,94) = 2.352, *p* = .064, *R*^2^ = .003), with no differences between the two training conditions (*F* (1,94) = 0.194, *p* = .330, *R*^2^ = .000). There was no significant improvement as a result of the two training groups in performance of the selective attention task, the subscales of the selective attention task, the paired-associates task, the reasoning task, and the memory span task (all *p* > .05). In addition, the two training groups did not differ in their training gains in the selective attention task, the subscales of the selective attention task, the reasoning task, and the memory span task (all *p* > .05). However, the two training groups differed with respect to their paired-associates task performance change (*F* (1,96) = 4.570, *p* = .018, *R*^2^ = .015), indicating a larger training gain for the simultaneous training group than the single cognitive training condition.

**Table 4:** Multiple regression for the interaction between orthogonal contrasts and linear trend for the cognitive transfer tasks

**Variable**	***B***	***SE***	**β**
Selective Attention			
Linear Interaction A × BC	0.52	0.59	0.08
Linear Interaction C × B	0.81	1.14	0.06
Paired-Associates			
Linear Interaction A × BC	0.04	0.09	0.03
Linear Interaction C × B	0.36	0.17	0.13*
Executive Control			
Linear Interaction A × BC	0.43	0.24	0.11*
Linear Interaction C × B	-0.20	0.47	-0.03
Reasoning			
Linear Interaction A × BC	0.04	0.14	0.01
Linear Interaction C × B	-0.09	0.28	-0.01
Memory Span			
Linear Interaction A × BC	-0.08	0.26	-0.02
Linear Interaction C × B	0.24	0.55	0.02
Processing Speed			
Linear Interaction A × BC	0.32	0.21	0.06
Linear Interaction C × B	-0.18	0.41	-0.02

*Notes.* A = Control Group, B = Single Cognitive Training Group, C = Simultaneous Training Group.

* < .05.

### Motor-cognitive dual task

At baseline, participants reduced gait velocity (*F* (1,58) = 82.469, *p* < .001, *R*^2^ = .587) and increased step-to-step variability (*F* (1,53) = 10.417, *p* = .002, *R*^2^ = .164) from single to dual task condition. There was a significant difference between the groups in velocity reduction (*F* (1,58) = 3.165, *p* = .05, *R*^2^ = .098) but not in increase of step-to-step variability (*F* (1,53) = 2.541, *p* = .088, *R*^2^ = .087). Both main effects of gait velocity and step-to-step variability for the factor group were not significant as well (*p* > .05). Considering the data of the interim test and posttest, participants still reduced gait velocity significantly but did not increase gait variability during dual task, with no significant main effect for group or interaction (all *p* > .05). Compared with the single task training and control group, the simultaneous training led to a significant reduction of the step-to-step variability during dual task (*F* (1,78) = 2.958, *p* = .045, *R*^2^ = .018) but not during single task walking (*F* (1,78) = 0.207, *p* = .326, *R*^2^ = .001), indicated by a significant interaction of the first contrast with the linear trend during dual task (Table 5). There was no different change as a result of the training between the single cognitive training condition and the control group with regard to gait variability change during dual task (*F* (1,78) = 0.073, *p* = .788, *R*^2^ = .000) and single task condition (*F* (1,78) = 0.459, *p* = .500, *R*^2^ = .003). Both gait velocities for single and dual task walking as well as the difference between dual and single task velocity did not change over the three points of measurement either for the simultaneous training compared to the single cognitive training and control group or the single cognitive training compared to the control group (all *p* > .05). There also was no larger improvement of the simultaneous training group in the working memory task in terms of correct calculation backwards or errors, with no differences between the single cognitive training and the control group in correct calculation backwards and errors as well (all *p* > .05). Motor-cognitive dual task performance is displayed in Table 6.

**Table 5:** Multiple regression for the interaction between orthogonal contrasts and linear trend for the motor-cognitive dual task

**Variable**	***B***	***SE***	**β**
Gait Velocity ST			
Linear Interaction AB × C	0.09	0.09	0.05
Linear Interaction B × A	0.09	0.16	0.03
Gait Velocity DT			
Linear Interaction AB × C	0.04	0.14	0.02
Linear Interaction B × A	0.35	0.26	0.06
Gait Variability ST			
Linear Interaction AB × C	-0.04	0.09	-0.04
Linear Interaction B × A	0.11	0.16	0.06
Gait Variability DT			
Linear Interaction AB × C	-0.05	0.27	-0.13*
Linear Interaction B × A	-0.01	0.49	-0.02
WM Correct Calculations			
Linear Interaction AB × C	-0.06	0.11	-0.03
Linear Interaction B × A	0.04	0.21	0.01
WM Errors			
Linear Interaction AB × C	0.14	0.12	0.09
Linear Interaction B × A	0.15	0.23	0.05

*Notes.* A = Control Group, B = Single Cognitive Training Group, C = Simultaneous Training Group, ST = Single Task, DT = Dual Task, WM = Working Memory.

* < .05.

**Table 6:** Performance of motor-cognitive dual task during pretest, interim test, and posttest

	**Simultaneous training (*N*** **= 18)**						**Single cognitive training (*N*** **= 12)**						**Control group (*N*** **= 21)**					
	**Pretest**		**Interim**		**Posttest**		**Pretest**		**Interim**		**Posttest**		**Pretest**		**Interim**		**Posttest**	
**Variable**	***M***	***SD***	***M***	***SD***	***M***	***SD***	***M***	***SD***	***M***	***SD***	***M***	***SD***	***M***	***SD***	***M***	***SD***	***M***	***SD***
ST Gait Velocity (m/s)	1.21	0.20	1.24	0.20	1.22	0.19	1.18	0.15	1.17	0.13	1.21	0.17	1.18	0.16	1.17	0.17	1.23	0.15
DT Gait Velocity (m/s)	1.00	0.17	1.07	0.25	1.07	0.20	1.07	0.18	1.12	0.16	1.12	0.20	1.02	0.18	1.08	0.17	1.12	0.14
ST Gait Variability	3.23	1.17	3.23	1.22	3.34	1.40	3.08	0.94	3.12	0.64	3.63	2.13	3.04	0.81	3.17	1.16	3.16	1.17
DT Gait Variability	4.89	3.18	3.80	2.11	3.38	1.02	3.44	1.56	2.78	1.22	3.28	1.04	3.47	1.60	3.62	1.65	3.53	1.31
Correct Calculations	3.33	1.94	4.11	2.37	4.17	2.20	4.33	1.47	6.27	2.00	5.45	2.70	3.58	2.58	4.18	2.04	4.53	2.85
Errors	0.83	1.15	0.83	1.92	1.00	1.14	1.00	1.00	0.27	0.47	0.64	0.67	1.06	1.14	0.82	0.95	0.88	1.22

*Notes.* ST = Single Task, DT = Dual Task.

## Discussion

The objective of the current study was to investigate training effects of simultaneously performed working memory training and physical training on cognitive and motor-cognitive dual task performance in older adults. It was hypothesized that the simultaneous performance of cognitive and physical training would lead to greater transfer effects in both single and dual task performance than single-domain training. Therefore, a simultaneous training group was compared with an active control group (cognitive training only) and a passive control group. Results showed that the participants of both training groups improved their performance substantially and comparably in the trained tasks over the course of the training. While both training groups improved their performance in the executive control task significantly compared to the passive control group, only the simultaneous training group demonstrated a significant increase in the paired-associates task in the course of the training when compared to the active control group. Furthermore, the simultaneous training group reduced its gait variability during dual task in the motor-cognitive task compared to both the active and passive control group.

Although overall training progress was observed in both training tasks, it was larger for the serial position training than the n-back training. This might have been due to the training material. Since the serial position task consisted of meaningful words, this type of training is rather susceptible to the use of strategies or mnemonics than the abstract n-back task with letters. However, there was no significant difference in the training progress between the two training groups. On one hand, this is somewhat unexpected, as older adults usually adapt to a motor-cognitive dual task by reducing the performance of at least one of the underlying tasks [44] and typically tend to prioritize walking and balance maintenance rather than the cognitive task [55]. As a consequence, this should affect the cognitive performance. However, in this specific situation, the participants could also have focused on the cognitive task, since they were secured with a safety belt and did not have to fear to fall. On the other hand, there might be a stimulating effect of treadmill walking on cognitive performance, which is consistent with other studies that demonstrated the immediate positive effect of moderate physical activity on cognition [33,34,56]. Therefore, physical activation could counteract the dual task-related reduction of cognitive performance. Regardless of the exact mechanisms, the similar training progress of both training groups makes the simultaneous training more efficient, as physical resources can be trained in parallel without negatively affecting the training progress in the cognitive task.

Even though the mean training progress did not differ between the two groups, the performance variance of the single cognitive training group over the course of both trainings tended to be larger, and the training performance in the simultaneous training group was more homogeneous. The physical activation could have helped to maintain concentration and attention during training, whereas individual differences could have become more evident during single cognitive training. In addition, the single cognitive training condition could have been perceived as more exhausting and less interesting and thus less motivating. In this case, the training potential of the single cognitive training actually would be greater at least for those who are motivated or able to keep focused, whereas the simultaneous physical activity is able to enhance motivation and promotes those who lack concentration or are exhausted after a certain time. Moreover, Sibley and Beilock [57] found only immediate positive effects of physical activity on working memory performance for those with poor baseline performance. As a conclusion, the simultaneous performance of two concurrent tasks may impair maximum cognitive performance, whereas the physical activity helps to improve performance at least to a certain level. A simultaneous cognitive and physical training then would be most indicated for those individuals with lower cognitive performance. In the theoretical framework for plasticity from Lövdén, Bäckman, Lindenberger, Schaefer and Schmiedek [43], acute physical activation may increase the range of flexibility for tasks that actually exceed the individual capacity limits.

Improvements from pre- to interim- and to posttest were observed in executive functions for both training groups. This is not surprising, considering that working memory capacity is an important component of executive functions and the constructs of working memory capacity and executive functions are highly correlated [58]. Therefore, the training gain in the executive control task can be interpreted as near transfer. However, there were no advantages of the simultaneous training over the single cognitive training. This is rather unexpected, since physical activity interventions usually show the strongest effect on executive functions [9]. Due to the strong correlation between executive functions and working memory, the potential training effect of physical activity or dual task performance could be interfered by the effect of the cognitive training. In this case, there is no additive or integrative effect of a simultaneous training on performance in tasks requiring similar abilities as the trained tasks. However, a recent study could only find effects of physical activity on executive functions for inhibition, but not for updating and shifting [59]. In line with this, different studies reporting on effects of physical activity on executive functions have only used inhibition tasks [11,13]. Therefore, the missing additional effect of the simultaneous training could also be the result of specific task characteristics.

The training groups did not improve their performance in the memory span task when compared to the passive control group. This is surprising, since this task was very similar to the serial position task that was trained. However, although the memory span task and the serial position training both aimed at remembering a series of words in the correct order, they still differed considerably. In particular, during the memory span task, the participants had to actively remember a series of words in the correct order, whereas during the serial position training, they had to asses whether the words in the presented word list were in the correct order or not. The similarity of those tasks with still different requirements could have caused interference.

Although there was no general training effect on the paired-associates task performance, the simultaneous training group significantly improved their performance when compared to the cognitive training group. This result is contrary to the results from the study of Fabre, Chamari, Mucci, Masse-Biron and Prefaut [30], who showed a general training effect for the combined and the mental training on paired-associates learning, but without any additional effect of the combined training. On one hand, our findings, therefore, possibly reflect the additional effect of the simultaneous training compared to a sequential or single training. On the other hand, the findings could also highlight the ineffectiveness of working memory training to improve paired-associates learning performance. In the study from Fabre, Chamari, Mucci, Masse-Biron and Prefaut [30], the cognitive training included a strategy learning of how to associate new information to a known reference point, which should definitively provide help in a following paired-associates learning task. Since in their study the physical training alone was able to improve paired-associates learning, the significant effect in the current study could result from physical activity alone. Unfortunately, given that the study design did not involve a single physical training group, this question cannot be answered with any certainty.

Performance in the other far transfer tasks such as selective attention task, reasoning task, and information processing speed task was unaffected by training. These results did not confirm the findings of other adaptive working memory training studies in old age [5,6]. However, training effects at least on reasoning ability in older adults are rather controversial. Whereas Jaeggi, Buschkuehl, Jonides and Perrig [60] found effects of adaptive working memory on reasoning in younger adults, they only found near but no far transfer effects of adaptive working memory training in older adults [7]. Moreover, in a recent study, von Bastian, Langer, Jäncke and Oberauer [61] found no transfer to reasoning ability neither in younger nor older adults. The missing additional effect of the simultaneous training is more difficult to interpret, since previous studies with combined training approaches did not include similar tests or summarized the results of the separated tests [30,31]. However, physical exercise alone has been shown to successfully improve at least attention and processing speed [10], but these results cannot be compared meaningfully, since we did not include a single physical exercise condition. As a consequence, different reasons could account for the missing training gain on the described tasks in our study. For instance, high baseline performance of participants in our study at least in the attention and processing speed tasks could have prevented any training progress.

According to our hypothesis, the participants of the simultaneous training group improved their performance in the motor-cognitive dual task from pre- to interim- and to posttest when compared to the participants of the two other groups. Moreover, only gait variability under dual task was affected, whereas variability under single task condition was not, indicating improved adaptability of the simultaneous training group to the motor-cognitive dual task condition. Dual task gait training has already been reported by previous studies, providing controversial results [62,63]. In these studies, training was either very similar to the criterion or transfer task or the studies failed to show transfer to new dual task walking conditions. The training condition in the current study differed considerably from the transfer task condition, even though both conditions include a walking and working memory task. One reason for the training effect could be the fact that the training was adaptive with continuously adjusted task difficulty, which keeps the training always demanding and should provide more training gains compared to non-adaptive trainings. As a result, this makes the simultaneous training of cognitive and motor components a more functional approach, as the simultaneous utilization of different resources often rather corresponds to the way they are required in daily life. Moreover, the current training approach could help to improve mobility and even prevent falls in older adults, as gait stability in the presence of an additional attentionally demanding task is more impaired with increasing age and related to increased risk of falls [64,65].

Although the findings of the current study are promising, one important limitation is the absence of a single physical training group that would allow for even more distinct comparisons. Ideally, the simultaneous training should additionally be directly compared to a combined training with sequential training conditions to clarify whether a simultaneous training group really performs better than a separated cognitive and physical training group. However, at least with respect to the motor-cognitive dual task performance, the simultaneous training should have clear advantages. Moreover, even if the simultaneous training was not superior to the sequential training, it is more efficient, since both components are trained at the same time, without impairing the training progress of the cognitive task.

A further limitation of our study is that the physical training was pulse-monitored and did not involve measures of individual fitness level before and after the training such as maximal O_2_ uptake (VO_2_max). Therefore, we could not determine whether participants’ cardiovascular fitness was really increased. Nevertheless, it is not clear if an increase of fitness is required for effects on cognition or increased cerebral blood volume for instance might be sufficient [20], which is already increased during moderate physical activity [23]. At least with respect to synergistic effects of simultaneous cognitive and physical training, increase of cardiovascular fitness does not necessarily represent a precondition.

Another limitation relates to the working memory training tasks, in particular to the serial position training, which was susceptible to the use of strategies. Training progress could at least to some extent be explained by individual differences in the development of successful strategies. Therefore, the use of strategies could have weakened the potential of the working memory training to transfer to other abilities, as strategy training usually only shows limited transfer [3]. Ideally, follow-up studies should consider adaptive cognitive training interventions not only with adjusting task difficulty but also alternating task requests to make the development of specific strategies even more difficult. In addition, the current participants have to be considered a highly selective sample with an active lifestyle and thus as not representative for the population at the same age. When training progress during cognitive task among those with low baseline performance is driven by additional physical activity, individuals with lower cognitive performance particularly should benefit from a simultaneous training, whereas those with already higher cognitive performance would not. Therefore, the effect of the simultaneous training in our study could have been underestimated. This factor could be addressed by investigating training progress depending on individual performance levels or among individuals with lower cognitive performance such as clinical subpopulations. Additionally, the number of participants in the current study was rather low. Therefore, there might be some difficulties to detect training effects in some of the transfer tasks. However, the specific contrast analyses based on the hypotheses should have adequate power to detect even small effects. This is reflected in the small effect sizes of the significant training effects in the present study. These small effect sizes could be the result of the rather short duration or intensity of the training with only two training session per week. To provide a stronger effect, the training may have to last longer or at least the intervals may have to be shortened. In addition to these rather small effects, the current results also do not provide any information regarding maintenance of the training effects and whether there are differences between the training conditions in terms of long-term maintenance. Future research is needed to determine long-term effects of simultaneous cognitive and physical training. Given the theoretical reflections of simultaneous training effects on neural activity and changes, it would be of great importance to investigate neuronal changes associated with simultaneous cognitive and physical training. Only this way, potential for cognitive plasticity can be completely examined.

## Conclusions

In conclusion, simultaneous cognitive and physical training was able to improve cognitive performance in the trained working memory task as well as in the executive control task, pared-associates task, and motor-cognitive dual task, whereas the single cognitive training only increased performance in the trained working memory task and executive control task. Therefore, the present results clearly demonstrate the potential of integrating cognitive and physical training programs to improve cognition and adaptation to situations requiring the recruitment of both cognitive and physical resources. The current results further indicate that trainings integrating different abilities should have greater effects on daily life functioning, which usually involves the recruitment of multiple abilities and resources rather than a single one. In addition, simultaneous training of cognitive and physical resources is more efficient, as both resources can be improved in parallel without affecting the training progress in the cognitive task. As a result, training programs should not only involve specific training of cognitive or physical resources to promote cognition in old age, but in particular simultaneous training of both resources.

## Competing interests

The authors declare that they have no competing interests.

## Authors’ contributions

NT: study preparation, acquisition of data, statistical analysis, interpretation of data, drafting manuscript. VS: study conception and preparation, acquisition of data, interpretation of data, revising manuscript. RA: acquisition and analysis of gait data. MM: supervision of study conception, interpretation of data, revising manuscript. LJ: interpretation of data, revising manuscript. All authors read and approved the final manuscript.
